# Fibroblast Growth Factor 21 Facilitates the Homeostatic Control of Feeding Behavior

**DOI:** 10.3390/jcm11030580

**Published:** 2022-01-24

**Authors:** Chih-Ting Wu, Aki T. Chaffin, Karen K. Ryan

**Affiliations:** Department of Neurobiology, Physiology and Behavior, College of Biological Sciences, University of California, Davis, CA 95616, USA; chtwu@ucdavis.edu (C.-T.W.); atchaffin@ucdavis.edu (A.T.C.)

**Keywords:** fibroblast growth factor 21, homeostasis, macronutrients, protein intake, sweet preference, neural mechanisms

## Abstract

Fibroblast growth factor 21 (FGF21) is a stress hormone that is released from the liver in response to nutritional and metabolic challenges. In addition to its well-described effects on systemic metabolism, a growing body of literature now supports the notion that FGF21 also acts via the central nervous system to control feeding behavior. Here we review the current understanding of FGF21 as a hormone regulating feeding behavior in rodents, non-human primates, and humans. First, we examine the nutritional contexts that induce FGF21 secretion. Initial reports describing FGF21 as a ‘starvation hormone’ have now been further refined. FGF21 is now better understood as an endocrine mediator of the intracellular stress response to various nutritional manipulations, including excess sugars and alcohol, caloric deficits, a ketogenic diet, and amino acid restriction. We discuss FGF21’s effects on energy intake and macronutrient choice, together with our current understanding of the underlying neural mechanisms. We argue that the behavioral effects of FGF21 function primarily to maintain systemic macronutrient homeostasis, and in particular to maintain an adequate supply of protein and amino acids for use by the cells.

## 1. Introduction

Food provides both energy and the organic building blocks needed for somatic growth, maintenance, and repair. Individuals must continually adjust their feeding behavior to balance supply with demand. A complex suite of neural and endocrine signals conveys information regarding energy needs and availability between the peripheral organs and the central nervous system [1,2,3] to maintain energy balance. Whether a comparable homeostatic system governs the supply and demand of individual macronutrients has been a topic of considerable interest and investigation [4,5,6]. Nonetheless, no substantial evidence has yet emerged in support of the homeostatic control of either fat or carbohydrate intake [7]. In contrast, accumulating evidence points to the homeostatic control of protein appetite. Although underlying mechanisms remain relatively unresolved, an important liver → brain neuroendocrine feedback control system, mediated by the hepatokine fibroblast growth factor 21 (FGF21), has very recently been described.

FGF21 is a liver-derived hormone that was discovered in 2000 [8], having pleiotropic effects on metabolic homeostasis. Excellent in-depth reviews have already described the several metabolic effects of FGF21 [9,10,11]. This review will instead focus on our understanding of FGF21 as a hormone regulating feeding behavior. First, we examine the nutritional contexts that induce FGF21 release, beginning with its original characterization as a starvation hormone and continuing to our current understanding of FGF21 as a signal of a low dietary protein-to-carbohydrate (P:C) ratio. We then discuss FGF21’s effects on feeding behavior and macronutrient choice. We argue that FGF21 primarily acts to maintain organismal proteostasis by increasing protein intake and decreasing carbohydrate and/or fat intake. Finally, we summarize our current understanding of the neural mechanisms by which FGF21 regulates this change in feeding behavior.

## 2. Nutritional Context(s) of FGF21 Induction

FGF21 is a polypeptide hormone secreted by hepatocytes. Although it can be found in several other organs, including the adipose tissue, skeletal muscle, and pancreas, the primary action of FGF21 in these tissues is autocrine or paracrine [12,13] (effects summarized in [9]). On the other hand, the endocrine action of FGF21 is regulated by the liver, which is overwhelmingly responsible for producing plasma levels of FGF21 in response to metabolic and nutritional challenges [14,15]. Therefore, we focus here on describing hepatic FGF21 as an endocrine signal of nutritional stress. Hepatic FGF21 expression and secretion is increased in response to disparate nutritional challenges, including starvation, a ketogenic diet, dietary protein restriction, and excess simple sugar or alcohol consumption.

### 2.1. Prolonged Fasting and Ketogenic Diet

FGF21 was originally characterized as a starvation hormone because it is elevated in animals subjected to a prolonged fast [16,17]. As glycogen stores become depleted, the main energy resource shifts from glucose to fatty acids and ketone bodies. FGF21 was initially described as a key hormone facilitating this metabolic shift [17,18]. According to this model [19], increased free fatty acids from adipocyte lipolysis circulate to the liver and activate the nuclear hormone receptor peroxisome proliferator-activated receptor α (PPARα). PPARα acts as a transcription factor that binds to the *Fgf21* promotor [16] and regulates its expression, and FGF21 signaling throughout the body inhibits carbohydrate metabolism and increases fat metabolism [20]. Multiple studies have shown that hepatic FGF21 expression is elevated after a prolonged fast, and that this adaptive response is ablated in PPARα-null mice [17,21]. Moreover, treatment with PPARα agonists such as fenofibrate and GW7647 increases *Fgf21* expression in mice [16,22] and humans [23,24,25]. The translational relevance of FGF21 as a fasting hormone is questionable, however, because circulating FGF21 is not reliably increased in human plasma until after a full 7 days of fasting [26,27,28], perhaps because rodents have a comparatively higher basal metabolic rate than humans [29]. Nonetheless, the data support that rodent and human hepatic FGF21 is enhanced during prolonged starvation, which is partly regulated by PPARα signaling.

A high-fat, low-carbohydrate ketogenic diet (KD) is another metabolic stressor that can increase circulating FGF21. In this model, similar to fasting, KD increases fatty-acid oxidation and ketogenesis, likely increasing the supply of endogenous PPARα agonists in the liver to elevate hepatic FGF21 [17,21]. In agreement with this, PPARα-null mice maintained on a standard rodent KD have markedly less *Fgf21* expression compared to controls maintained on KD. KD-induced FGF21 is not completely ablated, however, and increased circulating FGF21 is still detectable [17], suggesting that other transcription factors contribute to the rise in FGF21, as discussed below. Similar to fasting, the clinical translation of KD as a robust inducer of FGF21 is uncertain. Pharmacological activation of PPARα robustly induces FGF21 in humans [24,28,30], but the effect of KD in activating PPARα and increasing circulating FGF21 in humans is inconsistent at best [26,28,30].

### 2.2. Dietary Protein and Amino Acid Restriction/Dilution

Key recent studies have now resolved the above-noted translational discrepancies, by further defining endocrine FGF21 as a specific signal of dietary *protein* and/or amino acid restriction, rather than as a signal of starvation or ketogenesis *per se*. First, although hepatic *Fgf21* mRNA and plasma FGF21 protein were increased by about 3-fold after 24 h of starvation in rats, 12 h of re-feeding with pure carbohydrate or pure fat diets did not abrogate this effect. On the contrary, *Fgf21* was further increased, up to 12× that of *ad libitum*-fed controls [31]. A subsequent study [32] similarly found that rats that were food-deprived for 48 h had approximately double the plasma FGF21 levels compared to controls, and that re-feeding with a high-carbohydrate, low-protein diet dramatically *exaggerated* this response. Re-feeding with a high-protein, low-carbohydrate diet had the opposite effect [32]. Likewise, a deficit in dietary protein appears to mediate the FGF21 response to rodent KD [32]. Because mice and rats more efficiently use amino acids for gluconeogenesis, standard rodent ketogenic diets are not only high in fat and low in carbohydrates, but they also contain very little dietary protein. Rats and mice maintained on this KD exhibited high plasma FGF21, as expected, but importantly, this was not diminished by the addition of carbohydrates to the KD. Rather, the FGF21 response was abrogated by the addition of dietary protein [32]. These findings are consistent with a previous report that protein restriction, rather than excess fat intake *per se*, is the primary driver of hepatic FGF21 induction in KD-fed rats [33]. Lastly, dietary protein restriction also elevated plasma FGF21 in men, highlighting that this response to low dietary protein intake is conserved across species and resolving the clinical-translational discrepancy discussed above [32,34].

To better understand the mechanisms by which dietary protein dilution induces FGF21, several groups have investigated whether the increase in FGF21 observed during overall protein restriction can be recapitulated by restricting specific amino acids. FGF21 is increased following dietary restriction of branched-chain amino acids [35,36], sulfur-containing amino acids [37,38,39], all non-essential amino acids [34], and/or all essential amino acids [40]. FGF21 is also increased following the dietary restriction of several individual amino acids as well, including methionine, leucine, threonine, and tryptophan [40,41,42,43]. Finally, decreasing plasma alanine, asparagine, or glutamine via genetic manipulations in transgenic mice also causes increased FGF21 [34].

Mechanistically, it is well established that cells that undergoing amino acid deprivation activate the amino acid response (AAR) pathway. In this arm of the integrated stress response, an increase in uncharged transfer RNAs is sensed by general control non-depressible 2 (GCN2) kinase, which phosphorylates eukaryotic initiation factor 2α (eIF2α). eIF2α inhibits global protein synthesis, while simultaneously increasing the translation of specific genes involved in adaptation to amino acid deprivation, including activating transcription factor 4 (ATF4) [44,45]. De Sousa-Coelho and colleagues [43] identified *Fgf21* as a target gene of ATF4, revealing two ATF4-binding sequences upstream of the *Fgf21* human gene. Further data highlight the AAR as a key pathway of FGF21 regulation. GCN2-KO mice have significantly reduced basal expression of *Fgf21*, as well as blunted circulating FGF21 and hepatic eIF2a phosphorylation in response to a low-protein diet [46].

Despite a clear role in the regulation of *Fgf21* by the AAR pathway, at least two pieces of evidence point toward parallel and/or compensatory mechanisms for FGF21 induction. First, the increase in plasma FGF21 during acute dietary protein restriction was not completely ablated (although significantly blunted) in GCN2-KO mice [46]. Second, GCN2-KO mice maintained on a low-protein diet for over half a year showed progressively elevated FGF21 levels, eventually recovering *Fgf21* expression to the same level as the control group [46]. One alternate pathway involves PPARα, since PPARα-null mice exhibit remarkable decreases in plasma FGF21 when restricted of dietary protein, even for long periods of time. Interestingly, this occurs despite the continued activation of GCN2 → p-eIF2α [32]. Thus, dietary protein dilution can also activate PPARα → FGF21 independently of the AAR. One possibility is that low protein status relieves the inhibition of PPARα by mTORC1 [47,48], but this remains to be tested.

### 2.3. Simple Sugars and Alcohol

In 2009, Iizuka and colleagues [49] first showed, using rat hepatocytes, that glucose stimulates hepatic FGF21 expression through the transcription factor carbohydrate response element binding protein (ChREBP) [50]. When activated by intermediate metabolites of glucose and fructose [51,52,53,54], ChREBP binds the *Fgf21* promoter and increases its expression. Treating hepatocytes with simple sugars induces *Fgf21* mRNA expression [55,56,57]. Consistently with this, mice and rats consuming excess sucrose, glucose, or fructose, either acutely or chronically, exhibited increased activation of hepatic ChREBP and increased hepatic and circulating FGF21 [57,58,59]. The different magnitude and timing of this increase among the simple sugars glucose, fructose, and sucrose [57,60] was likely due to different gut → liver trafficking and differences in intracellular metabolism [61,62,63]. Importantly, the sugar-induced increase in circulating FGF21 was abrogated in liver-specific FGF21-KO and ChREBP-KO mice, supporting the notion that ChREBP regulates hepatic FGF21 secretion in response to simple sugar ingestion [57]. PPARα is also required for the ChREBP-mediated increase in FGF21, since PPARα facilitates ChREBP accessibility at the *Fgf21* promoter [64]. Importantly, and in contrast to the ambiguous translational relevance of fasting and ketogenic diets, independent studies have consistently demonstrated that excess intravenous and oral fructose and glucose increase plasma FGF21 levels in humans [60,65,66]. Robust induction of FGF21 does not occur in mice consuming similar amounts of polysaccharides and other complex carbohydrates [57], however, supporting a specific response to simple sugars rather than carbohydrates *per se*.

Circulating FGF21 is also elevated after acute or chronic alcohol consumption [67]. Mice acutely consuming alcohol significantly increase plasma FGF21 levels and hepatic *Fgf21* mRNA expression within a few hours [68]. This elevation also occurs and is sustained in mice drinking 30% alcohol for 16 weeks [69]. Consistently with these rodent studies, FGF21 is dramatically increased after binge alcohol consumption in humans [68,70]. Ethanol intake was reported to elevate ChREBP production and to raise FGF21 level in a similar time course as was reported for simple sugar intake [60,70], but the signaling pathways by which alcohol consumption increases FGF21 secretion are still undetermined and a potential mechanistic role for ChREBP in alcohol-mediated FGF21 secretion has not yet been reported.

Given the varying nutritional contexts that can induce FGF21 secretion, Solon-Biet and colleagues employed the Geometric Framework model to clarify the relationship between various metabolic contexts and FGF21 expression [36]. In this paradigm, over 800 mice were maintained on 25 different diets, varying in protein, carbohydrate, fat, and total calories. They found that a low-protein diet, especially when coupled with high carbohydrates, was the most potent inducer of FGF21 secretion. Neither fat intake nor total caloric intake independently predicted plasma FGF21 levels. Lastly, the geometric analysis showed that, whereas sucrose ingestion had no independent effect on FGF21 secretion, there was a synergistic interaction such that sucrose intake coupled with low-protein consumption provides the most effective stimulus for FGF21 [36]. The mechanisms underlying this synergistic (rather than additive) relationship are not fully delineated; it will be important to determine the potential contribution of molecular crosstalk among the various transcriptional regulators discussed above [GCN2 → EIF2α → ATF4; PPARα, mTORC1, and ChREBP] for this outcome.

## 3. FGF21’s Effect on Feeding Behavior

FGF21 is an endocrine signal of nutritional and metabolic stress (discussed above). Thus, its likely function is to act systemically to resolve this stress, thereby restoring physiological homeostasis. In this section, we discuss the mechanisms by which FGF21 may restore energy and/or macronutrient balance by acting in the brain to change feeding behavior.

### 3.1. FGF21 and Caloric Intake

**Figure 1:**
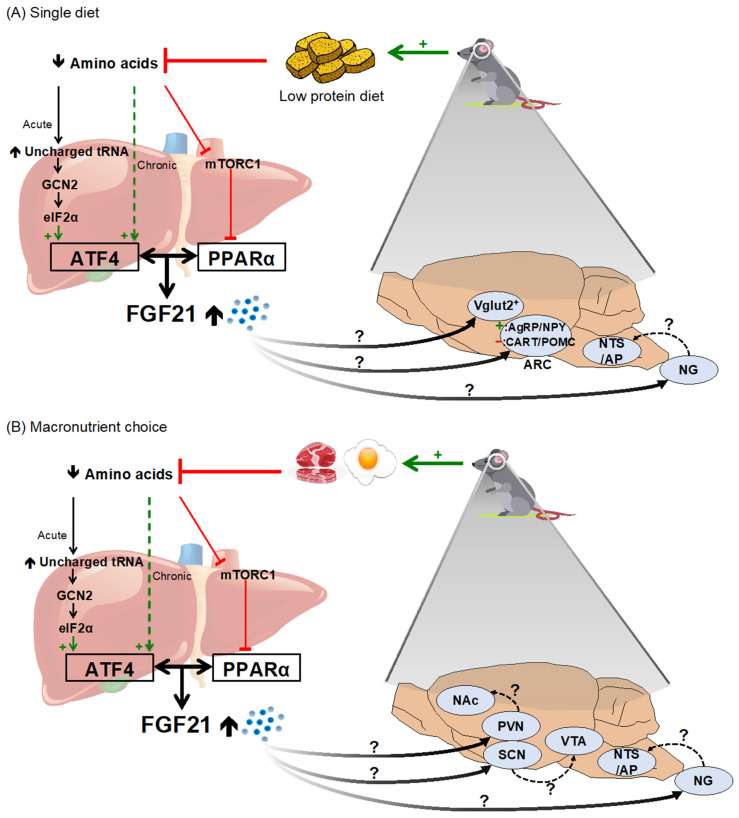
FGF21 and the regulation of (**A**) caloric intake and (**B**) protein consumption.

Physiologic, transgenic, and pharmacological activation of FGF21 signaling increases the total caloric intake in rodents. The initial publication describing the metabolic effects of FGF21 in 2005 [71] reported that transgenic mice overexpressing FGF21 in the liver consumed about 80% more calories compared to wild-type littermates. This FGF21-induced hyperphagia has been replicated in subsequent mouse genetic studies [72,73,74]. The increased caloric intake does not lead to obesity, however, because transgenic overexpression of FGF21 has a primary effect of increasing thermogenesis and energy expenditure, thereby causing overall weight *loss* [72,73,74]. Similarly, pharmacological administration of recombinant FGF21 has a dose-dependent effect of increasing total caloric intake, and also causes weight loss by increasing energy expenditure in mice [75]. Similar outcomes have been observed in many [76,77] but not all [78,79] rodent studies. The finding is more robust when caloric intake is calculated relative to total body weight, since pharmacological administration of FGF21 consistently increases energy expenditure and causes weight loss in male rodents [76]. Lastly, endogenous FGF21 is robustly induced by dietary protein and/or amino acid restriction (discussed above). In agreement with the pharmacological effects of FGF21, mice maintained on low protein and/or AA-restricted diets consume more total calories, together with increased energy expenditure and weight loss [32,34,80,81], and this depends on FGF21 expression in the liver [77,82] (Figure 1A). The effects of FGF21 on total caloric intake appear to be species-specific, since several groups have reported a decreased caloric intake in FGF21-treated non-human primates [83,84,85].

One (or both) of two likely mechanisms could explain the hyperphagic response to FGF21 observed in rodents. First, the increased caloric intake may represent a compensatory response, secondary to weight loss and increased energy expenditure [32,75]. Supporting this possibility, mice do not immediately change their caloric intake upon induction of FGF21. Rather, the hyperphagia occurs over the course of days or weeks, lagging behind weight loss by several days [72,79,86]. Second, the increased caloric intake may be secondary to protein leverage. The ‘protein leverage hypothesis’ [87,88] states that individuals regulate their intake of macronutrients such that protein intake is prioritized over fat and carbohydrate intake, causing excess total caloric intake when the diet is low in protein. This would be particularly apparent when individuals do not have the ability to choose among various food sources, as is common practice in laboratory rodent husbandry.

### 3.2. FGF21 and Macronutrient Selection

In addition to increasing total caloric intake, evidence supports a role of FGF21 in influencing macronutrient selection and/or sweet and alcohol intake when individuals are presented with dietary choice. First, two independent genome-wide association (GWAS) studies identified single nucleotide polymorphisms (SNPs) at the *Fgf21* locus that were associated with macronutrient intake in humans. Variants rs838145 and rs838133 were associated with low protein and fat consumption and increased consumption of carbohydrates [89,90] and variant rs838145 was also correlated with circulating levels of FGF21 [90]. Further analyses showed that rs838133 carriers were specifically associated with high intake of sweet foods [66]. A novel rs11940694 variant, located near the locus for FGF21’s obligate co-receptor, β-Klotho (*Klb*), was associated with alcohol consumption [91,92]. Based in part on the compelling associations between *Fgf21* and *Klb* genotype and food selection observed in these human populations, experimental studies using rodent and non-human primate models now support a mechanistic role for FGF21 in controlling sweet taste, alcohol, and dietary protein intake, detailed below.

### 3.3. FGF21 Increases Protein Intake

In 2019, two independent studies showed that FGF21 acts in the brain to increase dietary protein intake in male mice. First, our own laboratory used two complementary macronutrient choice paradigms to demonstrate that pharmacological administration of FGF21 causes a shift in macronutrient selection. Mice were first allowed to choose freely among three pure macronutrient diets (corn starch, casein, and vegetable shortening) for 4 days, establishing a baseline. Next, we delivered FGF21 or saline via i.p. injection and monitored the change in macronutrient selection over the following dark/active period. FGF21-treated mice showed increased casein and reduced starch intake, with no effect on fat consumption. To determine whether protein or carbohydrate was the primary macronutrient being regulated by FGF21, we next conducted a series of two-diet choice experiments. In each experiment one macronutrient was matched between two pelleted diet choices, whereas the other two macronutrients were varied. Importantly, sweetness was controlled across diets to avoid potential confounding effects. When mice were presented with two diets with the same fat content, FGF21-treated mice increased their percentage of kcal intake from protein and (necessarily) reduced the intake from carbohydrate. When mice were presented with two diets with the same carbohydrate content, FGF21-treated mice increased their percentage of kcal intake from protein and (necessarily) reduced the intake from fat. When diets were matched for protein, however, FGF21 had no effect on diet selection [77]. These results support that FGF21 increased protein consumption at the expense of either carbohydrates or fat. The FGF21-induced protein appetite was blunted when *Klb* expression was knocked down in the brain, showing that FGF21 signaling in the brain is necessary to shift macronutrient selection [77].

Consistently with this finding, Hill and colleagues (2019) showed that intracerebroventricular (i.c.v.) injection of FGF21 markedly increased the consumption of a 35% vs. 5% casein pelleted diet in a two-diet choice test. The investigators next maintained mice on low-protein or control diets for seven days and allowed individuals to choose freely between pure casein vs. pure maltodextrin solutions. Protein-restricted mice consumed more casein and less maltodextrin compared to controls. This behavior was attenuated in FGF21-null mice and in mice lacking *Klb* in neurons—demonstrating that FGF21 facilitates the homeostatic control of dietary protein intake via its actions in the brain [82] (Figure 1B).

In both studies [77,82], the effect of pharmacological FGF21 treatment on increasing total caloric intake was absent or blunted when treated individuals had access to dietary choice. That is, when mice were able to increase protein consumption by shifting diet selection, they no longer exhibited hyperphagia relative to saline-injected controls. These findings suggest that FGF21-induced hyperphagia may primarily be an attempt to maintain systemic proteostasis via protein leverage, rather than a compensation for increased energy expenditure, as discussed above.

### 3.4. FGF21 Reduces Sweet and Alcohol Preference

In 2016, two independent studies reported that FGF21 negatively regulates simple sugar intake and sweet preference in male mice [57,93]. Mice with high circulating FGF21, induced by either genetic or pharmacological approaches, exhibit decreased preferences for sucrose and non-caloric artificial sweeteners. For instance, when transgenic mice overexpressing FGF21 were offered a choice between a high-sucrose diet (HSD) vs. standard chow for several weeks, they consumed less HSD and more chow compared to wild-type controls [57]. Likewise, pharmacological treatment with recombinant FGF21 decreased HSD preference [57]. In two-bottle preference tests, FGF21 administration decreased the consumption of 3% and 10% sucrose and 10% glucose solutions compared to water [57,93]. FGF21 also reduced the consumption of the non-caloric sweeteners sucralose (10 mM) and saccharine (0.2%), relative to water [57,93]. Conversely, FGF21-null mice consumed more HSD compared to chow, and consumed more sweet solutions compared to water [57], confirming that endogenous FGF21 has a physiological role in sweet taste preference. The authors observed no effect of FGF21 on the consumption of maltodextrin (2%), intralipid (20%), maltose (100 mM), lactose (100 mM), liposyn (20%), casein (8%) (but see [77,82]), or quinine (1.5 mM), narrowing the influence of FGF21 to sweet tastes. FGF21 also reduces sweet consumption in non-human primates. Saccharine preference was markedly reduced in cynomolgus monkeys after administration of PF-05231023, a long-lasting analog of FGF21 [93].

Our own laboratory recently reported that recombinant FGF21 administration decreases sucrose intake and increases chow intake in male C57Bl/6J mice [77]. In agreement with the absence of an effect on maltodextrin preference observed by Von Holstein and colleagues [57], we found that FGF21 does not alter the preference for complex carbohydrates when sweetness is kept constant [77]. Thus, FGF21 decreased the consumption of sweet tastants, but it did not affect the consumption of carbohydrates *per se*.

FGF21 similarly decreases alcohol consumption. First, transgenic overexpression [93] or pharmacological administration [92] of FGF21 reduced ethanol preference in mice in a two-bottle preference test (4–16% ethanol vs. water). This decreased alcohol preference was blunted in mice lacking FGF21-receptors in CamKIIα-expressing cells, suggesting that FGF21 signaling in neurons is necessary for this effect [70,92,93].

**Figure 2:**
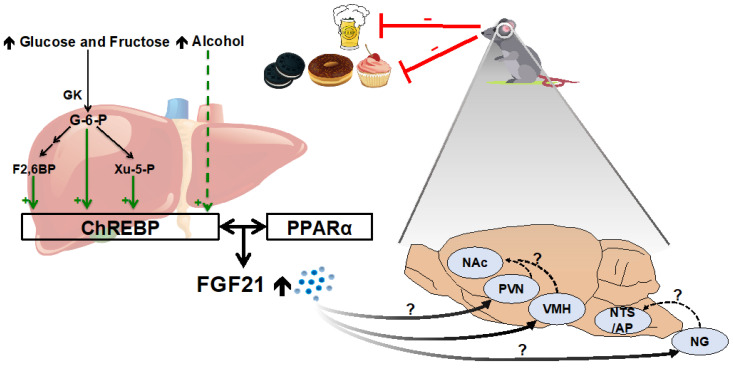
FGF21 and the regulation of simple sugar and alcohol intake. GK, glucokinase; G-6-P, glucose-6-phosphate; F2,6BP, fructose-2,6-biphosphate; Xu-5-P, xylulose-5-phosphate.

The findings discussed above raise an interesting possibility—that FGF21 directs the homeostatic control of sweet and alcohol consumption [94] (Figure 2). In such a model, excess consumption of simple sugars or alcohol elicits a physiologic and behavioral response, directed by FGF21, that reduces subsequent sweet/alcohol intake until a target value (or range of values) is reached, defending an equilibrium. Further support for this potential ‘equilibrium’ model is still needed, however, since sweet and alcohol intake are not otherwise known to be regulated via negative feedback control [5,6,95]. For example, do individuals that consume a large bolus of sucrose modify subsequent food choices to avoid or reduce the consumption of sweets? If so, does this behavioral change require FGF21? Future studies would be needed to directly address these questions.

## 4. Neuroendocrine Mechanisms for the Control of Feeding Behavior by FGF21

It is clear that the nervous system plays a crucial role in regulating FGF21’s effect on feeding behavior. FGF21 crosses the blood–brain barrier through simple diffusion [96], and its behavioral effects are significantly blunted in animals lacking FGF21-receptors broadly in neurons [57,77,82,93,97]. The logical next question concerns specific neuroanatomical substrates for FGF21 action. That is, *where* are the critical first-order neurons and *what* are the downstream mediators for FGF21’s effects on caloric intake and macronutrient selection?

### 4.1. Neuroanatomical Distribution of the FGF21 Receptor Complex

FGF21 signaling requires both the FGF receptor 1c (FGFR1c) and an obligatory co-receptor called β-klotho (*Klb*) [73,98,99,100]. *Fgfr1c* is broadly expressed throughout the body and brain, and when paired with heparan sulfate proteoglycans on the plasma membrane, it can be activated by several autocrine and paracrine members of the FGF family of signaling molecules. By contrast, endocrine members of the FGF family, including FGF21, have a loss-of-function mutation in the heparan binding domain and occupancy at FGFR1c is instead facilitated by β-klotho [98,99]. *Klb* is more discretely localized than *Fgfr1*, thereby determining the anatomical specificity of FGF21 action [98,101,102].

Progress towards understanding the neural circuit mechanisms of FGF21 action has been hindered by uncertainty about the neuroanatomical distribution of KLB. An initial whole-body FGF signaling atlas reported that *Klb* mRNA was expressed in peripheral metabolic tissues, with very low to no expression in sampled regions of the mouse brain [101]. Shortly thereafter, however, a more focused analysis from the same group, using laser capture microdissection, qPCR, and in situ hybridization, observed *Klb* to be highly and exclusively expressed in only three brain regions—the suprachiasmatic nucleus of the hypothalamus (SCN), the nucleus of the solitary tract (NTS), and the area postrema (AP)—and in the nodose ganglia (NG) [103]. In a subsequent publication, sparse but appreciable *Klb* mRNA was additionally observed in the ventral tegmental area (VTA) and nucleus accumbens (NAc) [93]. Meanwhile, both Liang and colleagues [104] and von Holstein-Rathlou and colleagues [57] separately reported KLB protein and mRNA (respectively) in the paraventricular nucleus of the hypothalamus (PVN). Jensen-Cody and colleagues later added the arcuate (ARC) and ventromedial (VMH) nuclei of the hypothalamus and the anterior piriform cortex (APC) to the list [97]. To provide clarification, Hultman and colleagues (2019) performed a comprehensive survey of *Klb* expression in whole mouse brains, using automated RNAscope in situ hybridization and droplet digital PCR, to better resolve the central anatomy of this system at the cellular level [105]. There the authors reported high-density *Klb* labels in the SCN only, with low-density labels in the reticular thalamus, medial trigeminal neurons, and the hypoglossal nucleus. Inconsistent labels, not above background levels, were reported in the PVN, the anterior (AH), dorsomedial (DMH), and ventromedial (VMH) nuclei of the hypothalamus, as well as the AP and the NTS. *Klb* was absent from other brain regions, including the APC, ARC, NAc, and VTA. Thus, the neuroanatomical distribution of *Klb* remains unresolved. Inconsistencies in the above studies may result from methodological differences, or may be influenced by internal and environmental conditions, e.g., nutritional status, time of day, sex, age, and social cues—which have not yet been considered.

### 4.2. Mechanistic Basis for FGF21’s Effect on Total Caloric Intake

Although the effect of FGF21 in terms of increasing caloric intake clearly arises from its action in the brain, the specific neurocircuit mechanisms are not yet known. Pharmacological, transgenic, and nutritionally-induced FGF21 increases energy expenditure by acting directly at its receptor complex in the nervous system, increasing sympathetic drive to the adipose tissues [32,72]. Thus, when *Klb* is deleted from neurons under control of the CamKIIα promoter (Klb^∆CamKIIa^ mice), FGF21 no longer increases energy expenditure or induces weight loss [72,82,103]. Mice lacking *Klb* in neurons also do not increase protein intake in response to FGF21 [77,82]. In agreement with this, the effect of FGF21 on increasing total caloric intake appears to be blunted in Klb^∆CamKIIa^ mice [82]. The phenotypic identity of these first-order neurons facilitating the hyperphagia is largely undetermined, but a recent study implicates the vesicular glutamate transporter. When *Klb* was deleted from *Vglut2*-expressing cells, the feeding response to dietary protein dilution was lost [106].

The ARC and PVN of the hypothalamus comprise a well-known circuit governing the homeostatic control of energy balance. In the ARC, FGF21 administration increases the mRNA expression of the orexigenic peptides agouti-related peptide (AgRP) and neuropeptide Y (NPY), whereas reducing or not affecting anorexigenic peptides cocaine and amphetamine-regulated transcript (CART) and proopiomelanocortin (POMC) [76,107,108]. However, whether these neuropeptides or their receptors are required to facilitate FGF21-induced hyperphagia is unknown. *Klb* mRNA has been reported in ARC in one recent study [97] but future work will be needed to determine if this is a direct site of FGF21 action, or if its effects on neuropeptide expression are indirect.

### 4.3. Mechanistic Basis for FGF21’s Effect on Macronutrient Intake

An initial report describing neurocircuit mechanisms underlying FGF21’s effect on sweet taste concluded that the first-order *Klb*+ cells facilitating this behavioral response are in the PVN [57]. The authors deleted *Klb* in specific hypothalamic regions by means of the stereotaxic injection of AAV-Cre directly into the PVN or SCN of *Klb*^flox/flox^ mice. Next, when *Klb*^∆^^PVN^ and control mice were treated with FGF21 and offered sucrose in a two-bottle choice test, the control mice showed the expected decrease in sucrose intake, whereas mice lacking *Klb* in the PVN did not. These mice also exhibited an increased HSD preference in a similar manner to FGF21-KO mice. On the other hand, *Klb*^∆^^SCN^ mice, and transgenic mice lacking *Klb* in *phox2b*-expressing cells (including neurons in the NTS and AP) responded similarly to controls.

Several years later, the model was further refined. First, the glutamatergic (*Vglut2*-expressing) population of *Klb*+ cells were broadly identified as critical mediators for FGF21’s effect on sweet taste preference [97]. Deleting *Klb* from *Vglut2+* cells, but not Gaba-ergic (*Vgat+*) or dopaminergic (*Dat+*) cells, using Cre-Lox mouse genetics, eliminated the effect of a 3-day FGF21 treatment to decrease the consumption of 10% sucrose or 0.2% saccharin vs. water. Conversely, activating *Klb* in *Vglut2+* cells using Designer Receptors Exclusively Activated by Designer Drugs (DREADDs), decreased sucrose preference [97].

Several neuropeptides that are produced in the PVN have been previously implicated in sweet taste preference, including oxytocin (OXT, [109,110]) and corticotropin-releasing hormone (CRH, [111,112]). Moreover, previous work had identified *Klb* colocalization in OT- [113] and in CRH- [104] expressing neurons of the PVN. However, deleting *Klb* in these neuronal populations did not diminish the effect of FGF21 to reduce sucrose intake [97]. Furthermore, when *Klb* was deleted from Sim1-expressing neurons, a commonly-used genetic model for whole-PVN deletion, the effect of FGF21 on sucrose intake was not changed [97], contradicting the conclusion drawn from the prior work (discussed above). Rather, more recent studies argue for the contribution of glucose-sensing *Klb+* neurons in the VMH [97]. Additional research will be needed to clarify these discrepancies and to identify downstream circuits and molecular mediators.

Both sweet tastants and alcohol activate VTA dopamine neurons and increase the dopamine release in the NAc. This dopaminergic projection is a common mediator for most reinforcing substances [114]. Moreover, glutamatergic input within the mesolimbic dopamine pathway influences both sweet [115,116] and alcohol [117,118] intake, raising interest in how FGF21 may interact with this system to control ingestive behavior. Two weeks of FGF21 administration significantly reduced dopamine and dopamine-related metabolites and increased the expression of the dopamine transporter in the NAc. Moreover, mRNA levels of catechol-*O*-methyl transferase, an enzyme degrading dopamine, were reduced in the VTA but not in the NAc after FGF21 treatment [93]. Another study revealed an increase in the dopamine release in the NAc after FGF21 treatment [107]. These data support a role of FGF21 in modulating dopamine signaling, but currently, the available data are only associative. Dopamine signaling that occurs acutely in response to a nutritional stimulus (e.g., sweet vs. neutral foods) is the critical variable thought to convey incentive salience and/or reward-prediction error, and it will therefore be most informative to discover how FGF21 modulates dopamine signaling acutely in response to specific food cues.

Although the effect of FGF21 in increasing dietary protein intake arises from its action in the brain, the specific neurocircuit mechanisms are not yet known. One interesting possibility is that FGF21 increases the reinforcing properties of dietary protein. Increasing the physiological need for amino acids is thought to increase the rewarding and/or reinforcing properties of protein, perhaps mediated by the mesolimbic dopamine system [119,120]. For example, Liu and colleagues (2017) demonstrated that in *Drosophila,* DA-WED neurons, the dopaminergic neurons projecting from protocerebral posterior medial 2 to “wedge” neuropils, encodes a persistent protein hunger in response to protein deprivation [121]. In adult rats, an increase in c-fos expression in the NAc was observed after high-protein diets were presented to animals previously restricted of protein, relative to non-restricted controls [119]. Moreover, a history of dietary protein restriction increased the evoked dopamine release in the NAc, compared to controls [122].

Dopamine reward prediction errors are shaped by physiological state [123]. Because FGF21 is a signal of protein deficiency and sweet/alcohol excess, it can be expected to modulate striatal dopamine signaling in response to specific food cues—increasing the dopamine response to dietary protein and decreasing the response to simple sugars and alcohol—leading to increased protein and decreased sweet and alcohol intake. This remains untested. Additionally, it is not known how the FGF21 signal may be conveyed to the mesolimbic pathway. Limited data support KLB expression directly in the VTA and NAc [93]. Otherwise, the information may be conveyed indirectly, for example, via an indirect circuit pathway to the VTA via the medial preoptic nucleus (MPON) [124].

## 5. Future Directions

As discussed above, FGF21 is secreted from the liver in response to various nutritional and metabolic challenges, including starvation, a ketogenic diet, excess consumption of simple sugars and alcohol, and amino acid imbalance or deficits. Accordingly, a rapidly growing collection of studies now support a novel role for FGF21, acting via the nervous system, in regulating feeding behavior by increasing caloric intake in single-diet-feeding paradigms, reducing the relative consumption of sweets and alcohol, and by increasing the relative consumption of dietary protein. Thus, like many hormones, FGF21 controls both physiology and behavior. These new findings prompt additional questions for future research, including:

(1) To what extent are the several behavioral responses to FGF21 unavoidably connected? That is, can the observed reduction in sweet and/or alcohol intake be dissociated from a compensatory increase in protein intake (and vice versa)? This has so far been difficult to disentangle experimentally—for example, in our hands decreased consumption of a sucrose solution following FGF21 injection was balanced by a corresponding increase in the consumption of chow [77]—but this could be addressed with a careful experimental design.
(2) What are the key neural circuit mechanisms mediating the effect of FGF21 on feeding behavior? Recent findings have identified critical neuronal phenotypes for sweet taste preferences (e.g., glutamatergic neurons of the VMH [97]), but some discrepancies remain unresolved, and downstream neural circuit mediators must be identified. The key first-order neurons for changing caloric and protein intake remain unknown, as is the extent to which circuits controlling caloric, sweet, and protein intake are intertwined.
(3) How does the nervous system integrate information conveyed by FGF21 with other well-characterized signals of energy status? For example, does FGF21 influence leptin and insulin signaling in the arcuate and elsewhere [125,126,127,128]; glucose sensing via glucokinase in neurons of the VMH [97] and elsewhere [129,130]; and/or amino acid sensing via mTOR or GCN2 in the mediobasal hypothalamus, hindbrain [131,132,133], and anterior piriform cortex [44]?
(4) What is the role of FGF21 in influencing the circadian control of feeding behavior? Plasma FGF21 follows a circadian rhythm that peaks early in the light phase and falls throughout the dark phase [134,135], and FGF21-transgenic mice have a dysregulated circadian pattern of locomotor behavior [103]. Since macronutrient intake is known to follow a circadian rhythm, with rodents favoring carbohydrate intake at the onset of dark and protein and fat intake at the onset of light [136,137], it is interesting to speculate about the potential role of FGF21 in facilitating this pattern.
(5) What is the therapeutic potential of these new findings? Because pharmacologic administration of recombinant FGF21 elicits multiple metabolic benefits in animal models, including decreased body weight, improved insulin and leptin sensitivity, and decreased hepatic steatosis, as reviewed by [9,10,11,138], several pharmaceutical companies have now developed FGF21 analogues for clinical use in metabolic disease [11,139,140,141]. One exciting possibility is that these drugs may also be useful to modify behavior. Potential applications include the treatment of alcoholism [142] or combating protein malnutrition and sarcopenia in aging [143].

## 6. Summary

The data discussed above strongly support a role for FGF21 in controlling feeding behavior in animals and humans. This work builds on initial human genome-wide association studies that linked genetic variants in the FGF21-receptor signaling pathway to differences in protein, sweet taste, alcohol consumption, and to our evolving understanding of the nutritional contexts for FGF21 secretion. FGF21 secretion is increased in response to amino acid deficits, and to simple sugar and alcohol excesses. Conversely, several groups have now demonstrated that pharmacologic, genetic, and physiologic induction of FGF21 increases protein intake, reduces the consumption of sweet foods and liquids, and decreases alcohol consumption. Such findings point to a special role for FGF21 as an endocrine signal mediating the homeostatic control of macronutrient intake with a special emphasis on maintaining systemic proteostasis. Given the importance of nutritional balance for health and well-being, a more complete delineation of the underlying intracellular and neurocircuit mechanisms may reveal novel targets for intervention in nutritional and metabolic health.
